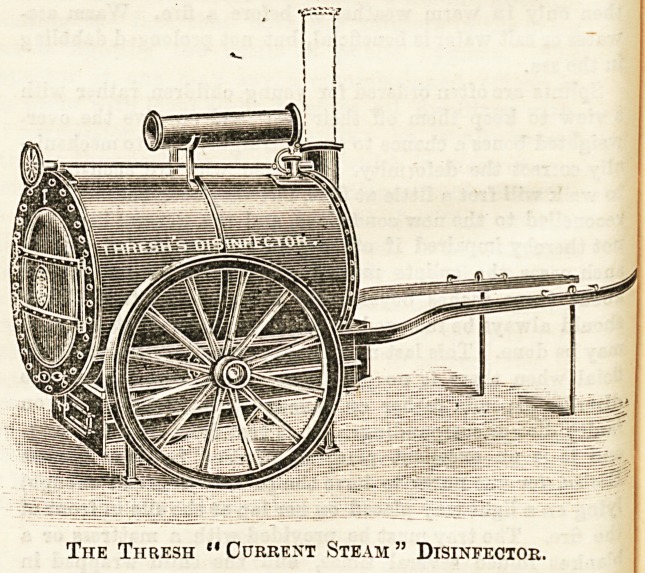# "The Hospital" Nursing Mirror

**Published:** 1899-03-04

**Authors:** 


### "The Hospital" Nursing Mirror.

Being the Nursing Section of "The Hospital."

[Contributions for this Section of "The Hospital" should be addressed to the Editor, The Hospital, 28 & 29, Southampton Street, Strand, London, W.C., and should have the word "Nursing" plainly written in left-hand top corner of the envelope.]

### News from the Nursing World.

### THE DUCHESS OF YORK AT KINGSTON.

On Friday afternoon the Duchess of York delighted the soldiers' widows residing at the Royal Cambridge Asylum, Kingston, by visiting them, and presenting each with a packet of tea and sugar. The value of the gift was enhanced by being made personally and accompanied by a friendly chat.

### LADY WARWICK AT ASTON.

Another of the many nurses' homes that have been established to commemorate the Diamond Jubilee was opened at Aston, near Birmingham, by Lady Warwick, on February 22nd. The fund collected for this purpose reached the handsome total of £4,191. Of this sum £860 has been expended in the purchase of Aston Villa Club, in the Albert Road, for the nurses' residence, and £2,550 has been invested in ground rents, which produce an income of £82 15s. A superintendent and two assistant nurses began work last September, but the formal opening was deferred until all arrangements were completed. The usual ceremonies attending such functions took place after the opening in the Council Chamber, when Lady Warwick was presented with a gold key. The Lady Mayoress of Birmingham and the Bishop of Coventry were amongst those present. The hon. secretary read the annual report, which was approved. The chairman then proposed a vote of thanks to Lady Warwick, who replied that it gave her real pleasure to assist anything for the promotion of nursing work. She was president of seven associations in different parts of the country, so that it would be seen that she was not quite ignorant of the working of such institutions. In recommending the Aston institution to the support of the public, she said many people she knew started with the conviction that it must be, if not a paying, at least self-supporting institution. But our hospitals exemplified the principle that the poor were utterly unable to pay for sick nursing. She also deprecated making philanthropy cheap and doubly dangerous by giving only a small amount of inefficient training to those who work amongst the poor. Such women cost less than a trained nurse, but it was hardly necessary to point out that if they wished to raise and improve the condition of the poor, and to teach them by example to live healthy and more refined and orderly lives, no nurse was too good for the work.

### SIOUX NURSES.

From one awakened conscience to a body of skilled nurses is a long step, and yet this is the history of the Sioux nurses. Not nine years ago a young doctor became a convert to the Roman Catholic faith, and entering the priesthood was stationed at the Indian Mission, Elbow Wood, North Dakota. Here he founded an order of American Indian Sisters, of which the first member was "White Dove," a daughter of the Sioux chief "White Hawk." She was a gentle, intelligent girl, and became in course of time the Reverend Mother Guertrude, and principal of a nursing school which grew so largely that at the outbreak of the Spanish-American hostilities no fewer than twelve sisters volunteered their services. Of these four were accepted, and are now at Havana with the Seventh Army Corps, under General Lee. By their English names they are known as Mother Bridget, Prioress General; Mother Anthony, Assistant General; Sister M. Joseph, and Sister M. Gertrude; Bridget being the very commonplace alternative of the poetical "Sound of the Flying Lance," which is the Prioress General's Indian name. Father Francis Craft, the founder of the sisterhood, under whose supervision the sisters have been trained, was injured in the battle of Wounded Knee in 1891, where also, as children, the twelve volunteers first experienced the horrors of war. The capable nurses at Havana had, therefore, an accurate and personal knowledge of the conditions under which they would work.

### HOPE CASTLE, ANTIBES.

Lady Murray's convalescent home for writers, on the Riviera, is appropriately named Château de l'Espérance. We gave a little account of it some time ago, and now the roomy villa standing in extensive gardens is open to jaded pen-folk of all nationalities. The great drawback to English visitors is, of course, the long and expensive railway journey, but the service of expresses from London to Nice is speedy and convenient, rendering the travelling not uncomfortable, whilst the moderate £1 a week for board and residence is very cheap indeed, and helps to reduce the cost of the change. There is only one route to Antibes (which is the station before Nice), and the return fares, first class, are £12 1s. 7d., and second class, £8 15s. 5d. Dr. Lunn's Italian Circular Tours to Genoa, however, cost a little less than the ordinary first class fare. Applications for admission must be made to the Hon. Lady Murray, Villa Victoria, Cannes. The length of visit is limited only by the necessities of the case. The home is closed from May 1st to November 1st every year.

### THE DISTRICT NURSES OF BELFAST.

Two points are brought prominently to notice by the twenty-fifth anniversary of the Society for Providing Nurses for the Sick Poor at Belfast. The first is the value of district nurses in helping to check an epidemic, and the second the practical way in which such societies can deal with the problem of old age provision when they seriously set themselves to the task. The annual meeting was held on February 22nd, at the Municipal Buildings, and the large attendance and numerous letters of sympathy expressed the public appreciation of the nurses' work. Typhoid was the most numerous of all diseases, and out of the 155 cases nursed only 17 died. One nurse contracted the disease and was well nursed in the fever wards of the Royal Hospital, and she also happily recovered. The occasion of the "Silver Anniversary of Our Foundation" is regarded as a fitting opportunity to commemorate the event by augmenting the superannuation fund for old nurses. Such nurses as are over 40 and unable to join the Royal National Pension Fund have been encouraged to invest £3 yearly in the Post Office Savings Bank by the Society adding another £3 to it; and nurses of the earlier days whose pay was wholly inadequate to make any provision for age receive pensions of from 3s. 6d. to 6s. a week. A liberal response to this appeal will enable these sums to be increased, and the scheme must commend itself to all who recognise the importance of each society charging itself with the care of those becoming aged in its service; and who value the public services of the district nurse.

### OPENING CEREMONY AT HULL.

The fund collected in Hull to form a memorial of the sixtieth anniversary of the Queen's reign was divided into two portions, the one to be spent on the erection of an art gallery, the other on a nurses' home. The nurses' home has been finished first, and was opened on February 21st by the Mayor (Councillor Gelder). How deficient Hull still is in the matter of district nursing may be estimated when (according to the calculation which assigns one nurse to every 10,000 of the population) 23 nurses ought to be engaged in this work, and there are only six actually employed! The new home affords accommodation for 12, nearly half the number needed. The empty purse, of course, lies at the root of the deficiency, and the sooner Hull awakes to the true economy of filling it the better for all. The Hull District Nursing Association dubbing itself anew the Hull Jubilee District Nursing Association, in honour of the event commemorated, has taken formal possession of the new quarters in Charlotte Street, which is situated centrally, and affords scope for the enlargement of the association's range of work. The cost has been £1,200 for the premises, with an additional £800 for alterations and furnishing. Owing to the comparatively small space at the disposal of the committee invitations were limited to members of the association.

### NURSES' HOME AT NEWCASTLE.

The conditions for the plans of the new infirmary for Newcastle-on-Tyne which is to be built out of Mr. John Hall's gift of £100,000 have been sent to eighteen architects. It will contain beds for 180 medical and 220 surgical cases. The nurses' home must afford separate bedrooms for 80 nurses, sitting-room and bedroom for the assistant matron, sitting-room and bedroom for the night superintendent, a reception-room for the nurses' private visitors, a large sitting-room and smaller writing-room for the sisters, two large rooms and a writing-room for the nurses and probationers. There must also be accommodation for the domestic staff of six housemaids. Each will be allotted a separate bedroom, and the servants' hall will also be fitted up for preparing tea. There will be eight bathrooms, and convenient store, cloak, and other necessary rooms.

### LEEDS DISTRICT NURSING ASSOCIATION.

Alderman Tetley, in the absence of the Lord Mayor, occupied the chair at the annual meeting of the Leeds District Nursing Association. Miss Garlick, the hon. secretary, read the twentieth report, which recorded an increase of work. The nurse who began lately in Armley has already more work than she is able to do, and steps have been taken to secure a second nurse to help her. In commemoration of the Queen's Diamond Jubilee a permanent nurses' home has been acquired. The purchase of the house and the adjoining premises in Lovell Street cost £3,100, their adaptation for their present use cost another £2,250 whilst the £2,500, remaining out of the £7,500 Jubilee gifts, has been banked. Part of the buildings have been let to good tenants, and the outlay, therefore, brings in a yearly rental of £125. The finances, however, are in an unsatisfactory state. For two years the association has been falling into debt, which now stands at £400. The committee have also before them several applications for nurses, which it is impossible to entertain unless the income is largely increased. The staff numbers 16 nurses, two superintendents, and the matrons; and, according to Mr. R. Benson Jowitt, who made the financial statement with regard to the Jubilee Fund, there is work for 30 in the city.

### DISTRICT NURSING AND SANITARY INSPECTION.

Speaking at the annual meeting of the Birmingham District Nursing Society, the Lord Mayor said that "he felt sure that it had only to be recognised by the men of Birmingham what an excellent work the society was doing for that work to be better supported. Recently they had been pointing to the necessity of having women inspectors in the health department, and he ventured to say that where the district nurses were engaged those inspectors would not be wanted." Everyone must agree with the Lord Mayor with regard to the district nurses' value as sanitary missioners. Nevertheless, they are not sanitary inspectors, and can never replace those necessary officers. The spirit and nature of the two spheres are widely different, notwithstanding the fact that both are soldiers in the army that is fighting the battle of public hygiene. The inspector is, one may say, a commissioned officer in this army with legal right to enter all dwellings without permission, and correct faults; he is also armed with the thunderbolts of the law which in cases of contumacy will be used to ensure obedience to his dictates. He (or she) is, moreover, trained for this specific task. The nurse, on the other hand, is a volunteer. She has no rights, no legal authority; she has had no special training for the work. Her skill is to administer to the sick human tenements; her right of entry is only that of an invited guest; her power to correct lies in kindness and persuasion; she is, in fact, the velvet glove, whilst the sanitary inspector is the iron hand. The two form a combination at once powerful and gentle, and admirably fitted to spur on and encourage the poor to rise up and banish from their home their own greatest physical enemies—dirt and disease.

### SHORT ITEMS.

The urgent necessity of providing a cottage hospital for Cupar, Fife, was discussed at the annual meeting of the Sick Poor Nursing Association held recently It was suggested that a house be obtained for the nurse with two rooms of two beds each available as a hospital; a committee was appointed to forward the project.—"A continued demand for the services of nurses" was reported at the annual meeting of the Peterborough District Nursing Association at Paisley. The staff numbers eight Queen's nurses, and many of the cases attended have been so severe as to necessitate more than one daily visit from the nurse.

### Hints on the Home Nursing of Sick Children.

By J. D. E. Mortimer, M.B., F.R.C.S., formerly Surgical Registrar, &c., at the Hospital for Sick Children, Great Ormond Street.

(*Continued from page* 223.)

### RICKETS—ACUTE RICKETS—INFANTILE SCURVY—PERITONITIS (ACUTE)—ABDOMINAL TUBERCULOSIS—DISORDERS OF NERVOUS SYSTEM.

### Rickets.

It is impossible in this place to discuss at length the causation and treatment of this so common disorder; but as it may have to be reckoned with in the management of any kind of illness, I must briefly allude to it. Special attention must be paid to the state of the digestion and the character of the evacuations, for the patient, although taking a good quantity of fresh milk, may be unable to assimilate it, and a special dietary may be needed. Pure air and sunlight should be freely admitted (of course with proper consideration), and the child should be in the open air whenever possible—provided there is no fog or east wind, and that care is taken by means of warm covering, hot-water bottles, &c., to counteract the feebleness of the circulation and liability to catarrh.

If there is much perspiration of the head at night it should be laid on fine flannel, changed as often as necessary, and if the legs are continually outside the bedclothes (which must not be too heavy), pyjamas must be worn, or a flannel nightgown pinned below the feet. The child should be handled very carefully in washing and dressing, as there is much tenderness of the bones. A baby suffering from rickets will often cry when approached merely because he is afraid he is going to be handled. Ricketty babies should not be nursed much in the arms nor encouraged to sit up or stand, but should lie down in a cot or little carriage—a light tray or shallow basket is useful for carrying them about. Sponging with cold water should never be done unless there is good natural reaction, and even then only in warm weather or before a fire. Warm sea-water or salt water is beneficial, but not prolonged dabbling in the sea.

Splints are often ordered for young children rather with a view to keep them off their feet (and so give the over-weighted bones a chance to grow straight) than to mechanically correct the deformity. Children who are accustomed to walk will fret a little at first, but they soon become quite reconciled to the new conditions, and the general health is not thereby impaired if other points are attended to. In such cases the splints must be well padded, and project about three inches beyond the soles of the feet. They should always be removed at night in order that massage may be done. This last-mentioned proceeding is most beneficial when there is no great tenderness, especially if the disorder depends rather on constitutional defects than on faulty diet or surroundings. It is best done after the bath, and it is an excellent plan, as advised by Dr. Symons Eccles, for the nurse to have the patient (if small enough) lying on a light tray placed on her lap as she sits in front of the fire. The tray must be provided with a mattress or a blanket folded several times, and the child wrapped in warm flannel, only the part actually in hand being at all uncovered. Gentleness is essential.

### Acute Rickets.—Infantile Scurvy.

In this disorder, more often seen in well-to-do families than amongst the poor, the blood is so much deteriorated that hæmorrhages occur, usually around the bones, and the gums become spongy and bleeding. The skin may become stained purple or yellow from the colouring matter of the blood working to the surface, as in a bruise, and the swellings caused by the extravasation may be taken for tumours or fractures of the bones—the latter especially, as there is extreme tenderness. In fact, in severe cases there may be actually numerous fractures, or rather separation of the shafts of the bones from the articular ends. The limbs lie helplessly, and infantile paralysis may be simulated. The infant must, therefore, be handled most carefully. Feeding should be done with a rubber teat or tube, and probably raw meat juice or potato soup will be ordered. The former is made by finely mincing fresh lean steak, and leaving it in an earthenware vessel for four hours with just enough water to cover it; then straining through muslin which should be well squeezed and wrung. The latter consists of four tablespoonfuls of sieved potato mixed with a little cold milk, to which half a pint is gradually added, and the whole simmered for twenty minutes.

### Peritonitis (Acute).

As this may come on as a complication in the course of other abdominal disorders, and also in certain diseases of the chest, such as empyema, the nurse should in such cases be on the lookout for its signs—the pinched expression of the face, the vomiting, the gradual distension, and particularly the extreme tenderness and the well-known position on the back with drawn-up knees, and rigidly contracted abdominal muscles. The management is as in adults.

### Abdominal Tuberculosis, with chronic peritonitis, ulceration of the bowels, and enlargement of the mesenteric glands, is mainly seen in children.

Tabes mesenterica is a term formerly applied to cases in which enlargement of the glands is prominent as compared with the other processes, but when mothers speak of "consumption of the bowels" they usually mean only a chronic, non-tubercular catarrh of the stomach and bowels such as occurs in improperly-fed babies. It is most important to give an easily digested, rather concentrated diet. Milk given in a crude form, and large quantity, is not well borne, the curd ferments, and the ulcerated bowels are painfully and dangerously stretched by the gas so formed. It should as a rule be peptonized or given with malted food. Whey, cream, jellies, fish, chicken, or underdone meat, carefully pounded and sieved, are suitable, and much care should be taken to have everything well prepared and well served. The abdomen should be bandaged with flannel. Pain may be relieved by gentle rubbing or by fomentations. A cradle may be needed to take off the weight of the bedclothes.

### Disorders of Nervous System.

Children are much more liable than adults to widespread and alarming disturbance of the brain and other parts of the nervous system from comparatively slight causes. This is especially true of those who inherit an excitable disposition and suffer from rickets; in such, delirium may begin even when the temperature is but slightly above normal.

### City Orthopædic Hospital.

A very amusing and clever dramatic performance was given on Tuesday, February 28th, at St. George's Hall, by the Strolling Players in aid of the funds of the City Orthopædic Hospital, which is endeavouring to raise a sum of £4,000 needed to defray the cost of partially rebuilding, which has been commenced and which was rendered absolutely necessary in order to improve the sanitary arrangements. The piece chosen for performance was "The Arabian Nights," by Mr. Sydney Grundy, and it was very brightly and spiritedly rendered. The hospital ought to benefit considerably from this entertainment.

### Antiseptics for Nurses.

By a Medical Woman.

### XXIV.—STEAM DISINFECTING APPARATUS.—*Continued.*

Reck's low-pressure disinfecting machine, which is made in Copenhagen, has a non-jacketed disinfecting chamber, and is one of the cheaper forms of apparatus and well worth notice. It is intended to work with current steam and at a low pressure. Steam enters a pipe at the top of the chamber and drives out the air through a tube at the bottom,

Reck's Disinfector.

and when the air has been removed the opening to the exit tube is partially closed and steam is driven into the chamber under pressure until a temperature of 220 deg. Fahr. to 235 deg. Fahr. has been reached, and this pressure is maintained for 20 to 30 minutes by an automatic arrangement. Since the temperature reached depends on the pressure, the machines are made of different strengths, according to the maximum temperature that may be desired, and the price of the disinfector depends on its strength. The apparatus is usually fitted with two doors. The process of disinfection is carried out as follows: The clothes to be disinfected are first dried and are then introduced on a trolley covered with a cotton fabric, supported by a screen of galvanised gauze, into the chamber, the clothes being in a wire-work and felt-lined basket, and the doors are securely closed. Steam is then admitted freely in the way described above for four or five minutes to expel the air in the apparatus, and a current of steam at a pressure of 1 to 2 lb. is then maintained for about 30 minutes by means of an automatic valve, and is then shut off and a spray of cold water, called the "cleansing shower," is turned on for three minutes, entering at the top and falling over the umbrella-like covering of galvanised wire gauze and cotton fabric attached to the basket containing the clothes, and to which reference has already been made. By this means the water is distributed over a large surface, but the clothes are completely protected from being wetted by the dripping of water from the top of the apparatus, as there are no means of replacing the heat which has been lost by radiation through the walls of the chamber owing to the absence of a steam-jacket, &c. As a result of the spray of cold water, the remainder of the steam is condensed in the chamber, and a partial vacuum is consequently formed into which air rushes through an automatic valve. The clothes when removed are said to be quite, or very nearly, dry, but, as a matter of precaution and routine, they are shaken out to remove any remaining vapour and spread for a short time on a shelf in the "drying-room," which is heated from below by the "exhaust," or waste steam, from the disinfector. The special features of this are, the use of a low-pressure current of steam, delivered to the chamber by an automatic regulator at a rate which cannot be exceeded; absence of steam jacket, and the cold shower of water. Moreover, in the top of the disinfector is placed a plate on to which cold water is sprayed at the end of the operation, so as to rapidly condense the steam which is left in the apparatus after the disinfection is completed, and there is a hinged valve in the door of the apparatus which allows the entrance of air when a partial vacuum has been formed by the spray. Instead of removing the clothes on to a shelf for the purpose of drying them, if preferred an apparatus can be obtained having a special arrangement which can be attached for drying the clothes within the disinfector by means of a current of hot air. The disinfecting chamber can be had of any shape preferred, viz., round, oval, or rectangular, and also of any size required. The entrance and exit doors of the chamber are made steam-tight by means of bolts and screw nuts, and the whole apparatus may be had either arranged to be stationary or mounted on wheels, so as to move it from district to district as wanted. Where steam boilers exist it is only necessary to run a pipe from the boiler to the disinfector, which is already provided with its own pressure regulator. If there is no such boiler, a small one is provided with the apparatus, and, if space is limited, this boiler can be fixed beneath the disinfecting chamber. It is claimed that no skilled labour is necessary either to put up or to work this machine. Reck's disinfector has been subjected to very careful tests, and has been proved to be very efficient. As a result of experiments

The Thresh "Current Steam" Disinfector.

it was found that the temperature reached within 16 folds of blanket after 35 minutes was 200 deg. Fahr., and that anthrax spores within 16 folds of blanket were completely sterilised, and that the increase in weight of the goods after treatment was small. It has now been proved conclusively that a current of steam accomplishes disinfection in the most rapid, simple, and effectual manner, and consequently this method is employed in all the modern machines.

In Thresh's steam disinfector current steam is also used without pressure, and hence this is another of the machines which combine the greater cheapness and simplicity of construction which can be obtained when steam under pressure is not used. It has long been a well-known physical fact that the steam given off from water which contains substances in solution has a boiling-point proportional to the specific gravity of the solution, and that the temperature of the steam given off at the atmospheric pressure has a temperature several degrees higher than that of boiling water, and this fact has been utilised in Dr. Thresh's apparatus. The disinfector consists of a chamber, usually oval, supplied with jackets, and having a door at each end built into the brickwork and heated by a suitable furnace. The jacket is filled two-thirds with saline solution, and the steam obtained from this is first of all made to pass along a coil of pipes which are placed in the saline solution, and then it enters the chamber at the bottom and passes through the articles to be disinfected and finally escapes by a chimney. Moreover, when the steam is not being passed through the chamber it is all to escape freely into the chimney. The water that is lost by evaporation is replaced by means of a small tank, which is fitted with a ball-cock, by which the supply of water is regulated, so that it is kept at a constant level in the tank, and so constantly and steadily supplies the loss. The time needed for disinfection is about thirty minutes, this being counted from when the steam is first turned on into the chamber. After this time the valve which admits the steam is shut, and a valve at the lower part of the chamber is opened, and air is thus admitted, which is drawn up through a coil of pipes circulating in the saline solution into the chamber by means of the draught caused by the chimney. This current of warm air which is allowed to act for 30 minutes, together with the radiation of heat from the jacket, dries the disinfected articles very thoroughly and effectually. In this apparatus the temperature obtained is apt to vary to some extent with the rate of steam produced, and as there is no pressure gauge to record it a thermometer has to be provided to indicate the temperature, and the steam production regulated accordingly; but this method does not give as great a control as does a recording gauge, which is a great drawback to an otherwise excellent apparatus. The efficiency of a steam disinfector depends upon the steam not getting below a given minimum pressure, and without a self-recording gauge there is always the danger of a fall of pressure being overlooked by the attendant, and in consequence of the temperature being lower than what is necessary for efficient disinfection, or should there be a great press of work there might be a temptation to begin operations before the pressure was sufficient, all of which would be prevented by a self-recording gauge.

### West-End Hospital for Diseases of the Nervous System.

The Duchess of Devonshire reopened the wards of the West-End Hospital for Diseases of the Nervous System on February 28th. Amongst those present were Canon Porter, Lady Darrell, Sir Lennox Napier, and General Turner. Her Grace arrived at noon, and was received by the chairman, Mr. G. E. Porter, the hon. treasurer, Captain H. A. Dowell, and the senior physician, Mr. T. Outterson Wood, M.D., M.R.C.P. The Chairman, in a somewhat long speech, sketched the history of the hospital for the last twenty-one years, from the early days when the Princess of Wales consented to become patroness until the present time, when fifty beds were available for paralysed and nervously-afflicted children.

### Our American Letter.

The scheme for building a new nurses' home and training school at the Hartford Hospital is progressing favourably. Already, although it is only a short time since the project was mooted, some £3,000 of the £7,000 needed has been forthcoming, and the plans will soon be ready for publication. The new home will consist of three storeys. Forty-eight of the fifty bed-rooms will be in the two upper storeys, and the area of each will be 9 ft. by 13 ft. On the ground floor will be arranged the reception and dining rooms, and library and lecture room. The staff now numbers forty, and accommodation will be provided by these new premises for ten more. The period of training, which is now for two years only, will be then increased to three.

Most of the nursing associations are rejoicing in letters from members who have taken part in the exciting, if somewhat trying experiences of the last few months. One writes from the Military Hospital, San Juan, Porto Rico, of the impossibility of making one dollar do the work of five, and of the out-of-date notions of neatness possessed by the former occupants of their present quarters. The climate, too, has been damp and trying, so that, in spite of the romance attaching to such service, the hardships are not only very real but wearisome.

In December last the nurses of the Central Dispensary and Emergency Hospital, Washington, D.C., had the pleasure of taking possession of their new home, which is the gift of Miss Phœbe A. Hearst. Many eminent persons were present at the opening, and expressed themselves delighted with the excellence of the arrangements.

It is the intention of the Government to send at least a hundred nurses to Cuba. They will be placed with the division hospitals of the Seventh Army Corps. The services of the detachments already sent must have been valuable during the late disturbances.

Influenza has been very prevalent this season, and not a few nurses have suffered from it. In some institutions it has caused great inconvenience, as supplementary help has had to be engaged. Typhoid fever also has claimed more victims from amongst the volunteer nurses to the sick soldiers, but others are recovering and resuming their work again.

It is rumoured that a Bill will be presented to Congress for the establishment of what is really an army nursing corps, separate from the army medical corps. The idea of nurses rejoicing in the military titles is not a little ludicrous, and calls to mind the nomenclature of the Salvation Army. However, what's in a name? General Nurse Blank may be an excellent organiser, who will see that the dollars are spent profitably, and that the nursing camp is in readiness for the wounded, and who will not leave the wounded to wait for the nurses.

### Death in Our Ranks.

We regret to announce the death, on February 26th, of Nurse Widdicombe from typhoid fever, which developed after recovering from an attack of scarlet fever. She had been nursed through the former illness at the Boro' Hospital, and was recruiting her health at her own home, at Brixham, South Devon, when she again fell ill. Nurse Widdicombe entered the Portsmouth Royal Hospital as probationer in 1896, and after two years' training was attached to the private nursing staff. Her kind and gentle manners made her a great favourite with her patients, and her loss is greatly lamented by all.

### A Book and its Story.

"TO ARMS."

THERE is no lack of excitement in Mr. Balfour's last novel.* The scene of the story is laid partly in Scotland and partly in France during the first Jacobite rebellion, and the stirring events of the time give the author material he uses to the best advantage. The story opens on Tweedside, where we find Allan Oliphant, a lad and the future hero of the tale, living with his father and mother in what had been in former days one of the old border strongholds.

To the family party are presently added Henry Gering, an Englishman, who comes as tutor to Allan and Dorothy Wayward, a singularly fascinating girl, who comes on the scene somewhat mysteriously, and whose identity is not disclosed until the end of the book. Both the lad and his tutor fall in love with the girl, and finally, when her affection is given to Henry Gering, they come to fisticuffs about her in a scene which shows Allan at his worst and Henry Gering at his best. Gering then goes to seek his fortune elsewhere, and Allan presently sets out, against his father's wishes, to study medicine in Edinburgh. With his start for the Scottish capital the excitement of the book commences, and from this point until the tale closes we are led on from one adventure to another.

Reaching Edinburgh after the city gates are closed for the night our young hero falls in with the villain of the plot —a truly melodramatic scoundrel who subsequently appears in many disguises, and always when he is least expected. On the present occasion he makes Allan drunk and robs him. Allan finds his way into Edinburgh the next morning, and almost the first person he comes across is the most delightful character in the book, "Pittendreigh the godless," with whom he strikes up a friendship which lasts till the close of the tale.

Pittendreigh, an old man when he is first introduced to us, is still in swordsmanship, endurance, and agility, to say nothing of spirits, in his first youth. After many years as a soldier of fortune he lives mainly on the proceeds of some terrible pills he has invented—"Pittendreigh's Purifying Pills, as sold to princes, peasants, poets, philosophers, pikemen, and petticoats." "They are near as big as sugar plums, and of a fine red colour." Thanks to Pittendreigh's good offices Allan obtains a situation with the apothecary who manufactures these peculiar boluses, and, thanks to this employment, meets Dr. Elliot, the professor of anatomy at the college, who makes him his prosector. At his work in the dissecting-room he again comes across the villain who is to shadow him through the remainder of the tale. Dr. Silas Solid is what he calls himself in his present disguise. In reality a Hanoverian spy, he represents himself to be a London physician studying the methods of the Edinburgh medical school.

The description of Dr. Silas Solid as given by Allan, who writes the story in the first person, is hardly flattering: "I bowed as Dr. Elliott spoke, and looked the man up and down, and as I live I could scarce suppress a shudder, so cold, so passionless, so colourless did he seem to be. And yet there was nothing peculiar in his figure. He was about the middle height and of a very ordinary build, although perhaps his shoulders were a trifle squarer than is usual for a man of his bulk, and his hands and feet were so large as to be out of all proportion to his body. He was lean rather than stout, and the skin of his neck had a scraggy look, lying in lurks and wrinkles, and being of a dull yellowish-white colour, as though it were half jaundiced. It was his face, however, that fascinated me. . . . It was a long and clean-shaved face, coming sharply to a point below and not ill-shaped. He wore no wig, and his hair was very light in colour, of a shade like bleached straw, and, though fairly long, it seemed dry and brittle, and stuck out beyond the collar of his coat like the edge of a flesh brush. His forehead was long, low, and deeply wrinkled, and his eyebrows were scarce marked at all, the hairs composing them being of a lighter colour than even those of his head. His eyelashes had more yellow than white in them, and shaded but ill as strange a pair of orbs as ever looked out of human skull. They were close-set, and of that very pale and shifty blue which is seen in pigs' eyes that are not pink, while there lurked in them a cold, hard glitter very horrible to see, and yet which drew your gaze as the magnet draws the needle. His nose was long and sharp. . . ."

Shortly after the arrival of Dr. Solid Allan becomes the eye witness of a murder in the street at night, a man being shot under his eyes by apparently the same man who had robbed him on his first arrival in Edinburgh. Finding himself in a false position, Allan fears being charged with the murder, and takes to his heels, pursued by the watch, and finds sanctuary in a house occupied by Henry Gering. He escapes all suspicion, however, and returns to his work at the college, where Silas Solid persuades him to attempt "body snatching" to provide the dissecting room with a subject. He is successful in this enterprise, and examining the body finds that it is the corpse of the man he had seen shot. Whilst making a post mortem examination with Dr. Solid Allan comes across a curious ring in the body, which the Doctor endeavours to secure. This ring, it appears, is a secret one, containing concealed under the stone orders for the Jacobites, and it was to secure the possession of this jewel that Dr. Solid had shot the Jacobite emissary, who in his dying moments had swallowed it.

Allan is tricked into paying Dr. Solid a visit at night, and the latter discloses himself as Sir Oliver Wayward and the father of Dorothy. Allan escapes from Solid's clutches only to be seized by a party of Jacobites in mistake for Dr. Solid, and hurried away later to the Earl of Mar, and as a prisoner sees the drawn battle of Dunblane. Fate then throws him in contact with Prince Charlie, and he remains attached as a surgeon to the rebel army until the Pretender gives up all hopes of success and sails for France. Now, at the time of his being kidnapped Allan has made a mortal enemy of one of the rebels, Captain Le Fanu, and the latter succeeds in again capturing him and smuggling him over to France, where he imprisons him in the terrible prison of Henri Grenouille. Flooded out of his subterranean dungeon he manages to escape into the river, and is cast on shore at the villa of Madame de Verney, whose husband is a prisoner in the Bastille. With great boldness Allan succeeds in rescuing Monsieur de Verney, only to take his place in the prison. Here his surgical knowledge stands him in good stead, and he is befriended by the celebrated Monsieur Law. The young prisoner performs a delicate operation on a friend of M. Law with great success, and he is given his liberty and becomes the most successful surgeon in Paris, where he accumulates much wealth. Dorothy Wayward again appears on the scene, and he escapes with her to England, pursued by Dr. Solid. Dr. Solid is eventually hanged, and the book closes with Allan's engagement to Dorothy.

Such is a bald sketch of the episodes in this exciting book. From first page to last it is full of interest and sensation. The author has spared no pains to make his work historically correct, and if we may find fault it is that the coincidences are too frequent, and that the principal characters in the book appear in different situations and localities with too great regularity. But in spite of this the book will not willingly be laid down by anyone who commences it.

* "To Arms." By Andrew Balfour. (London: Methuen and Co. 1898. Price 6s.)

### Our German Letter.

By Our Own Correspondent.

A new field of labour for female nurses will ere long be opened up in the Taunus Bergen. One of the most important of the many public buildings which have been erected in various parts of those beautiful mountains is the Heilstälte Rupershaim. This well-known institution is among the oldest Volks hospitals in Germany, and no other foundation of the kind has done a greater or a nobler work. Unfortunately, however, it was designed for the accommodation of male patients only, and until a few weeks ago it seemed improbable that the dissatisfaction to which this circumstance has naturally given rise would ever be removed. Then a quite unexpected event took place. A Frankfurt family, desirous of raising a monument to the memory of a deceased relative, came forward with a donation of £10,000, which they have handed over to the committee of management on the distinct understanding that the money shall be devoted to the erection of a hospital for women, and which they have promised to supplement by an annuity to defray the cost of lighting the place by electricity, and by the sum of £1,250 for the endowment of one of the beds. These are to number from thirty-six to forty in all, and the need for the new hospital is so great that, if funds are forthcoming to maintain them, it is more than likely that the whole of them will be applied for long before the completion of the building, which will probably be opened in the autumn.

The current year may also witness the opening of two important additions to the municipal hospitals of Berlin. One of these is to be specially appointed for the reception of lunatics, but they are both to be erected on the same plot of ground at Buch, and the premises are to include but one church, but one dispensary, and but one bakery, the Council having wisely resolved to devote the money which the duplication of these departments would have compelled them to expend to the housing of their nursing staff. The married nurses, in particular, will benefit greatly by this decision. They are to be furnished with separate dwelling houses, in which they will be permitted to reside with their families, and which it is proposed to construct on a scale of quite unusual comfort and elegance. This is the more worthy of note, as the two existing municipal hospitals of Berlin are entirely without such accommodation for married members of their nursing staffs, who, indeed, are by no means so satisfactorily housed as they might be.

Preparations for the great conference on tuberculosis, which is to be held in Berlin at Whitsuntide, are proceeding apace, and the organising committee are arranging what bids fair to be a most interesting programme for the occasion. *Inter alia*, they have succeeded in securing a promise to attend from Dr. Rudolf Virchow, and the famous professor has consented to address the delegates on "Tuberculosis in its Relations to Food." The proceedings will extend over a period of four days, and invitations to be present are, I understand, to be sent to most of the leading authorities on tuberculosis.

Speaking of the congress reminds me that the third annual meeting has just been held of the Deutsche Zentral Komitee zur Errichtung von Heilstätten für Lungenkrank. This institution is under the patronage of the Kaiserin, and among its members are some of the foremost German physicians of the day. Its business, practically speaking, is to control the numerous unions started over here for the purpose of erecting hospitals for persons suffering from diseases of the lungs. During the past twelve months the number of such societies has been brought up to 33. Of these, eight have already established hospitals, and ten others will, it is expected, be able to erect similar establishments before the close of the present year. It is the special desire of the Zentral Komitee to provide for the treatment of women. At the twenty different hospitals for Lungenkranken which are now open in the Fatherland, the great majority of the beds are reserved for male patients; and accommodation for female sufferers of the same class is sorely needed everywhere.

One of the most useful institutions in Germany for the training of married and single women as nurses of the sick is the Bromberg Vereinigung der Freiwilliger Krankenpflegerinnen, and the latest annual report of this organisation is a document of extraordinary importance. For other than German members of the nursing profession, however, the only interesting paragraph it contains is that which refers to the Krankenpflegehilfsstelle which the union established, with the permission of the authorities, early in 1898. The Krankenpflegehilfsstelle is a sort of minor hospital for the very poor, and it affords the more advanced members of the Bromberg Vereinigung a splendid opportunity of putting the knowledge they acquire in the lecture hall into practice. During the past twelve months they spent 127 whole days and 82 whole nights at the place, nursing no fewer than 126 indigent patients there, 69 of whom were suffering from internal complaints; and their consequent progress is credibly reported to have been in the highest degree satisfactory. Work of the kind indicated could not, indeed, fail to be of the greatest value, and it is gratifying to learn that it is to be continued.

### Minor Appointments.

Kasr-el-Ainy Hospital, Cairo.—Miss Edith Coxon (whose appointment in February as Sister of the above hospital was given in our issue of February 18th), after receiving her training at the West Kent Hospital, Maidstone, became charge nurse at the Samaritan Hospital for Women, sister of the children's wards, Golden Square Throat Hospital; she also undertook the duties as sister during holidays at Great Ormond Street, and of matron-assistant West Kent Hospital, Maidstone.

Bath Statutory Hospital for Infectious Diseases.—Miss Josephine Whittaker was appointed Nurse-Matron of the above hospital. She was trained at the North-Eastern Hospital for Children, at the Plymouth General Hospital, and at the London Fever Hospital, at which institution she subsequently became sister. She was also sister at the Nottingham Isolation Hospital, and afterwards was attached to the staff of the London and Brighton Nursing Association.

Gosport and Alverstoke Isolation Hospital.—On February 10th Miss Charlotte Courtnell was appointed Matron-Nurse of this institution. She received three years' training at the Portsmouth Royal Hospital, and she has been successively charge nurse and head nurse of the children's ward and acting matron of the Fever Hospital, Milton.

Royal Berks Hospital.—Miss Ada Hunter was appointed Assistant Matron on February 21st. Miss Hunter was trained at the Royal Berks Hospital, where for the last two years she has held the appointment of theatre sister. She will now combine these duties with those of her new office.

Dawlish Infirmary.—On February 14th Miss Thompson was appointed Nurse-Matron of this infirmary. She was trained at the General Hospital, Birmingham, and has been matron of Sir Titus Salt's Hospital, Saltaire, and night superintendent of the Burton-on-Trent Hospital.

The Infirmary, Burton-on-Trent.—On February 2nd Miss Agnes Kirkpatrick Norie, who was trained at the Infirmary, Oldham, was appointed Charge Nurse of this institution.

Longton Cottage Hospital.—Miss S. E. Barlow has been appointed Head Nurse here. She was trained at the Stanley Hospital, Liverpool, and has been special nurse of the Jessop Hospital for Women, Sheffield.

### Workhouses or Almshouses.

By Miss Louisa Twining.

It is a remarkable coincidence that the discussions of the recent Poor Law Conference and the debate in the House of Commons should have occurred so simultaneously, and it is to be hoped that both will be considered by those who took part in them. It has been supposed that the day of "almshouses" is passed, and their place at present taken by an increasing adoption of the plan of pensions, on which persons may live with their own belongings and according to their respective likings. It is, therefore, somewhat surprising to find the system being now advocated as suitable for adoption by the State, and the suggestions are surely remarkable, for it appears that men and women are to be separated in households of ten, and apparently these all must be single or widows and widowers. The workhouse, at any rate, allows couples over 60 to live together in married quarters. It strikes one that those who brought forward the various suggestions can have little practical knowledge either of present arrangements or the classes for whom they would legislate; and if the new almshouses are to be paid for by the rates, what difference will be perceived between them and the workhouse? Again, who is to nurse and tend the sick and aged? Surely a "trained nurse," such as is now ordered in workhouses, cannot be expected in every house or cottage? One cannot help asking, is not the obvious and common-sense remedy to be found, as is suggested in your article, by the extension of classification, as already being carried out by many Boards, and also, as I would further suggest, by giving up the name "Workhouse," now wholly inapplicable, and reverting to the far more appropriate one of "Poorhouse," which would include all who would be entitled to enter it. It has been long urged that the amalgamation of so many and various classes under one roof and one management is an anomaly that is attempted in no other country than our own, and after sixty years of trial it is not surprising if the system is found to be wanting in adaptation to present times and circumstances. To separate the sick and aged in institutions corresponding to the "Hospices" of France, where "trained nursing" would find no difficulties and no "friction," and to leave the able-bodied, the casual, and the vicious classes to other management, would surely solve the problem of "Poor Law reform" in a way that nothing else will do.

### Everybody's Opinion.

[Correspondence on all subjects is invited, but we cannot in any way be responsible for the opinions expressed by our correspondents. No communication can be entertained if the name and address of the correspondent is not given, as a guarantee of good faith but not necessarily for publication, or unless one side of the paper only is written on.]

### AFFLUENT BREADWINNERS.

"Red Hackle" writes: Are there not other ennobling capacities where the gratuitous services of women of such self-abandonment as "Another Who Loves Nursing" would be inestimable blessings and where they would not be crowding out conscientious and intelligent, though less fortunate, women, were they inspired absolutely by that "holier side" referred to and not merely in quest of fame and romance as so many affluent breadwinners are? "Inasmuch as ye did it unto one of the least of these My brethren ye did it unto Me," relates to other friendly offices than ministering to the sick. I for one would be grieved to see the retirement of gentlewomen from the nursing profession, but is it not equally grievous that their affluence is being a direct means in excluding even talented poor women from a sphere at once congenial and remunerative? So many vacancies, both public and private, now stipulate that the candidates should be gentlewomen or ladies. Personally I define either terms "of gentle birth." Then for the obscure woman who merely has gentle acquirements there is no chance at all.

### A Poor Law Nursing Question.

The Chelsea Board of Guardians are to be congratulated on the final upshot of their lengthy discussions with reference to the selection of nurses, and that by a considerable majority they have adopted a reasonable resolution.

When the splendid work of the pioneer women guardians is remembered, it is the more regrettable that in so many instances their successors display small knowledge of those subjects which it should be their special province to study and understand.

The following unworkable resolution was proposed by a lady guardian (Miss Grove) at last week's meeting of the Chelsea Board (we quote from the *West London Press* of February 24th): "That there be a nursing committee appointed to deal with all candidates for workhouse and infirmary, to select for approval by the Board. That the clerk advertise in the usual way and hand all applications over to committee. Further, that the committee deal with all resignations of nurses for report to Board. Committee to consist of three ladies and three gentlemen, members of the Board, and that all previous resolutions relating to the appointment of nurses be rescinded."

Miss Grove further suggested an alternative course whereby the selection of candidates should be left to the "two matrons," though what the matron of the workhouse can be supposed to have to do or to know in connection with the nursing of the sick in the infirmary is hardly clear to an average intelligence.

An amendment was subsequently moved by Mr. Blore. "That as vacancies occur in the nursing staff the matron be requested to select candidates for the approval of the Guardians, and that the resolution of June 1st, 1898, and all previous resolutions relating to advertising for nurses be rescinded." After a great deal of wholly irrelevant talk a further amendment, embodying the principle contained in Mr. Blore's amendment, was proposed by Mr. Egerton, and ultimately passed by a large majority of those present. This amendment provided "That as vacancies in the nursing staff occur the matron be requested as far as possible to select two or more candidates for each vacancy as it occurs, and bring them before the General Committee for final decision."

The trend of the discussion seemed to show that the members of the board did not discriminate between the terms "selection" and "appointment." It cannot be too plainly understood that the two are in no sense synonymous. There is no question of making the matron responsible for the appointment of charge nurses; that power rests with the Guardians. The contention is that the matron, as a trained expert, is the proper person to select candidates for appointment by the Board, or, in other words, that the Board should appoint upon the matron's recommendation. It is not a matter of judging by testimonials solely, as some Guardians appear to think; many things besides the possession of certificates have to be considered, and it is foolishness to suppose that a lay committee, knowing nothing whatever about nursing and nurses, can be competent to decide upon the qualifications of applicants. As the *Chelsea Mail* rightly remarks, "Foremen select their necessary subordinates, not because they are independent of their employers, but because their employers are dependent upon their superior technical skill." There is undoubtedly an important principle at stake, a principle which lies at the root of good administration, and it is most satisfactory that the Chelsea Board of Guardians have taken the only right course in this matter, and thereby set an excellent example. The chief credit must be given to Messrs. Brass, Blore, Thomas, and Riley, and the Rev. Lawson Forster.

### The Book World for Women and Nurses.

[We invite Correspondence, Criticism, Enquiries, and Notes on Books likely to interest Women and Nurses. Address, Editor, THE HOSPITAL (Nurses' Book World), 28 & 29, Southampton Street, Strand, London, W.C.]

NURSING: ITS PRINCIPLES AND PRACTICE. For Hospital and Private Use. By ISABEL ADAMS HAMPTON. Revised and enlarged. Illustrated. (Philadelphia: W. B. Saunders. 1898. Price 7s. 6d. net.)

THIS new edition of Miss Hampton's work will be welcomed by nurses, not only because of its general merits as a handbook of nursing, but because it gives the reader a good deal of information about the routine in American hospitals. It is, indeed, something more than a mere handbook of nursing, for it also deals with the very different question how to teach nurses. The first chapter is on training school organisation and management, giving an outline of the course which should be followed in arranging the lectures and the examinations for a two years' and a three years' course respectively. It will be noted, on reading the chapter on the hospital ward, that the grades of nurses in America seem to be somewhat different from what obtain in some of our larger London hospitals, there being no "sister" over the ward, but in place of her a "head nurse," and that there is no second grade or charge nurse, the different nurses doing each sort of work in turn. The staff for a ward of twenty-five to thirty beds is described as consisting of one head nurse; one temperature nurse, who takes temperatures, gives medicines, keeps the medicine chest in order, gives out meals, and is responsible for the appearance of the kitchen; a nurse on the right side of the ward, who also looks after the linen closet; a nurse on the left side of the ward, who also looks after the bath room and lavatory; a third nurse takes care of the special patients in the small rooms, and is responsible for the preparation of the patients for operation; a probationer or junior nurse, who assists in making beds, cleans mackintoshes, looks after the clothes, and helps in giving meals. In addition, of course, there is a ward-maid, and in the men's wards it is to be noted that the ward-maid's place is taken by an orderly, who among other duties has to bathe the convalescents. Passing to the chapter on disinfectant solutions and their preparation, we note the extent to which the metric system of weights and measures is employed, and also observe that where the English measures are used the quart is taken, according to the Apothecaries' measure, as containing only 32 ounces, which makes the preparation of the familiar 1 in 20 or 1 in 40 solutions a somewhat complicated affair. It is curious in how many ways people are influenced by the weights and measures which they are in the habit of using. Probably the twenty ounces in the English pint has had much to do with the strengths of our solutions—one in twenty, forty, sixty, and so on. There are a good many remarks on the subject of douching and catheterisation which show a carefulness worthy of imitation. "Each patient in the hospital requiring douches should have her own douche nozzle. Before being used for another patient the nozzle is to be washed thoroughly with soap and cold water, and boiled for five minutes in a 1 per cent. solution of carbonate of soda." In passing the catheter in women free exposure is advised, with the previous sterilisation of the nurse's hands with perchloride solution and cleansing of the parts with boric solution and sterilised gauze pads before the catheter is inserted. Everywhere we see a careful recognition of the necessity for asepsis. "When thermometers are not in use they should be kept in a glass filled with a fresh solution of bichloride of mercury (1-1,000)." For the preparation of gauze drains it is recommended that the gauze should be cut "by drawn thread," a matter of some importance, which we have often seen overlooked, with the result that the drain is apt to leave shreds when it is withdrawn. A useful chapter is devoted to obstetrics, giving the nurse a short outline of what is necessary to do in cases of confinement, and we need hardly say that here, as throughout the rest of the book, the strictest care in regard to asepsis is inculcated. Chapters are given on special nursing required in various diseases, and finally a vocabulary of medical and other terms completes what is a very useful and complete nursing handbook.

THE GOOD REGENT. A Chronicle Play. By Professor Sir T. GRAINGER STEWART. (London: William Blackwood. 1898. Price 6s.)

To anyone whose taste inclines them to the intellectual we would commend this play. It is extremely interesting and admirably worked out; one rises, after its perusal, better versed in the study of the times when the Good Regent played so important a part in the making of the history of a country which at that time was at the height of its romance. We speak, of course, of the great religious transition period before John Knox became a household word in Scottish homes. Of the subject of his poem, the writer reminds his readers how diversely has James Stewart, Earl of Moray, Regent of Scotland, been judged. Warm supporters and bitter opponents have not been found wanting. Censure and praise alike followed in his train. Mr. Froude declared of this grave and reticent leader of the "precise Protestants" that in his opinion the Regent Murray was the honestest man in the whole island—but there was much pitch which he could not help handling. "The Good Regent" figures in lives of Queen Mary and in lives of John Knox, as in other histories of the period, but it has, so far, been left to Sir T. Grainger Stewart to present him as the chief figure on a canvas, so as to show his character in fuller light. "And," says the author in his preface, "I cannot but hope that those who give his case a fair hearing may find themselves in agreement with the views I have formed." Besides being interesting in itself, the play which is now before our notice has an educational value of its own, and shows the writer to be an earnest student of the times of which he writes.

### Royal British Nurses' Association.

MRS. GARRETT ANDERSON'S sessional lecture to the members of the Royal British Nurses' Association at 11, Chandos Street, W., was well attended by an appreciative audience. The subject was "The History and Effects of Vaccination," and the chair was taken by Sir THOMAS SMITH, Bart., who made a few remarks at the conclusion. The lecture, which was profusely illustrated with large diagrams hung round the room, will be published by Mrs. Garrett Anderson herself. She therefore made a point of asking that no notes should be taken for publication.

Country nurses enjoy the institution of tea and coffee, which can now be obtained prior to the serious business of the evening, and which lends a social friendliness to these reunions.

### Appointments.

LONGTON COTTAGE HOSPITAL.—Miss Mary Lambert has been appointed Matron of this hospital. She was trained at the North Riding Infirmary, Middlesborough, and has been matron of the Carmarthen Infirmary for three years, and of the Southport Infirmary for seven. Miss Lambert was elected out of 85 candidates.

### For Reading to the Sick.

"THE MIGHTY COMFORTER."

**Verses.**

THE world's a room of sickness, where each heart
Knows its own anguish and unrest;
The truest wisdom there and noblest art
Is his, who skills of Comfort best;
Whom by the softest step and gentlest tone
Enfeebled spirits own,
And love to raise the languid eye,
When, like an angel's wing, they feel him fleeting by.
*Keble.*

I only saw how I had missed
A thousand things from blindness,
How all that I had done appeared
Scarce better than unkindness.
How that to comfort those that mourn
Is a thing for Saints to try;
Yet haply God might have done less,
Had a saint been there—not I.
Alas! we have so little grace,
With love so little burn,
That the hardest of our work for God
Is to comfort those who mourn. —*Faber.*

I could not do without Thee,
I cannot stand alone,
I have no strength or goodness,
No wisdom of my own;
But Thou, beloved Saviour,
Art all in all to me!
And perfect strength in weakness
Is theirs who lean on Thee. —*F. R. H.*

I have a life with Christ to live,
But ere I live it, must I wait
Till learning can clear answer give
Of this or that book's date?
I have a life in Christ to live,
I have a death in Christ to die,
And must I wait till Science give
All doubts a full reply?
Nay, rather while the sea of Doubt
Is raging wildly round about,
Questioning of Life and Death and Sin,
Let me but creep within
Thy Fold, O Christ! and at Thy feet
Take but the lowest seat,
And hear Thine awful voice repeat
In guiltless accent, heavenly sweet,
"Come unto Me and rest;
Believe Me and be blest!" —*J. C. Shairp.*

**Reading.**

"But the Comforter, which is the Holy Ghost, whom the Father will send in My name, He shall teach you all things, and bring all things to your remembrance, whatsoever I have said unto you."—*John* xiv.

Christ's presence with His disciples was a blessed thing, and His absence would be a blank. Yet there was to be a substitute or successor; one who would comfort them in the Master's absence, and carry on His instructions, bringing the old to remembrance, yet adding new of His own. . . . Thus He is "the Comforter." He has been so; is so; and will be so until the Lord come. Have we used Him as such? Have we partaken of His fulness? Have we tasted the abundance of the everlasting consolation which He administers? or do we try to be our own comforters? Do we seek human comforters? Do we try our sorrows? or do we take all to Him, acknowledging His name and mission, and rejoicing at all occasions and opportunities for employing Him as the comforter? How much we lose by not going to Him, using Him as such.—*H. Bonar.*

### Notes and Queries.

**The contents of the Editor's Letter-box have now reached such unwieldy proportions that it has become necessary to establish a hard and fast rule regarding Answers to Correspondents. In future, all questions requiring replies will continue to be answered in this column without any fee. If an answer is required by letter, a fee of half-a-crown must be enclosed with the note containing the enquiry. We are always pleased to help our numerous correspondents to the fullest extent, and we can trust them to sympathise in the overwhelming amount of writing which makes the new rules a necessity.**

**Every communication must be accompanied by the writer's name and address, otherwise it will receive no attention.**

*Nursing Homes.*

(228) Could you give me any idea where there is an opening for a nurses' institute and nursing home, one on the sea coast preferred?—*E. A. (Surrey).*

It is impossible for us to recommend openings for nursing homes. What we advise is that nurses should have nothing to do with such ventures until they know from personal experience (1) that they are successful in the management of such institutions; (2) that they have found the right place for them to succeed in.

*Co-operation.*

(229) Could you tell me if there is a nurses' co-operation society in Glasgow or Edinburgh, and if so the address in either city?—*Brabazon.*

There is only one nurses' co-operation managed by nurses for nurses in London; but there are private institutions which pay nurses their earnings after deducting a percentage for expenses. The only one in Glasgow mentioned in "Burdett's Official Directory" is the Hillhead Nursing Institution, 66, Craigmaddie Terrace, Sandyford Street, Glasgow.

*Redress.*

(230) You so kindly help others, will you help me? I am a sanatorium nurse, and gave one month's notice February 1st. During the week I had an offer of an engagement, but permission was declined for me to go out about it, although I have not been off duty for five minutes for three weeks. To-day a case of German measles has been brought in, which will effectually prevent me seeking or taking another engagement on March 1st, when I leave. I have gone to the expense of buying numerous papers, and have advertised. What redress have I?—*A Sanatorium Nurse.*

This is a question for a lawyer.

*Superintendent's Post.*

(231) I am anxious to obtain a post as lady superintendent or matron in an institution for private nurses or in a convalescent home. I have been nursing eleven years.—*M. T. W.*

Watch the advertisements in the "Mirror" closely. Write and ask the matrons under whom you have served if they will kindly bear your wish in mind, and forward it if possible.

*Nubian Fast Black Lining.*

(232) In response to an inquiry from a correspondent, we have to state that the address of the wholesale agents for the Nubian Fast Black Lining is 12, Wood Street, E.C. This lining can be obtained, we understand, at Peter Robinson's in Oxford Street, at Garrould's in Edgware Road, as well as at nearly all the leading drapers.

*A Telescope Bed Support.*

(233) I am anxious to make known "a telescope support" to assist patients to keep up in bed which I have invented, and which Mr. Bailey, of Oxford Street, has made. Could you kindly tell me how to proceed?—*Nurse E. H. Y.*

The only way is to advertise in either THE HOSPITAL, *Lancet*, *British Medical*, &c., and to try and induce doctors to make use of the appliance so that it should gradually become known. Unfortunately, however, advertising is expensive.

*Diabetic Diet.*

(234) Will you kindly tell me if there is any reliable book on diabetes giving diet, &c., suitable for very poor person?—*M. H.*

"The Art of Feeding the Invalid" (Scientific Press, 3s. 6d.) is valuable for diabetic patients.

*Curable Paralysis.*

(235) Will you kindly tell me if there is any hospital that would take in a poor girl for treatment for paralysis of one leg and foot, as it is not considered to be a hopeless case?—*Anxious.*

The National Hospital for the Paralysed and Epileptic, Queen Square, Bloomsbury, W.C., admits patients free by letter of recommendation. Write to the secretary.

*Child Nurse Training Schools.*

(236) Would you kindly let me know the name and address of the institution where girls are trained as nursemaids?—*G. B.*

The Norland Institute, 29, Holland Park Avenue, Notting Hill, W., is the best known, though there are many other institutions now which devote attention to this subject.

**ANSWERS REQUESTED.**

(237) Can you suggest an antidote which country mothers may keep and apply for the bite of a poisonous snake known in the north (where it abounds in many of the woods, as in those near Broughton-in-Furness) as the hag worm. Dogs have been known to die from the sting.—*E.*

## Figures and Tables

**Figure f1:**
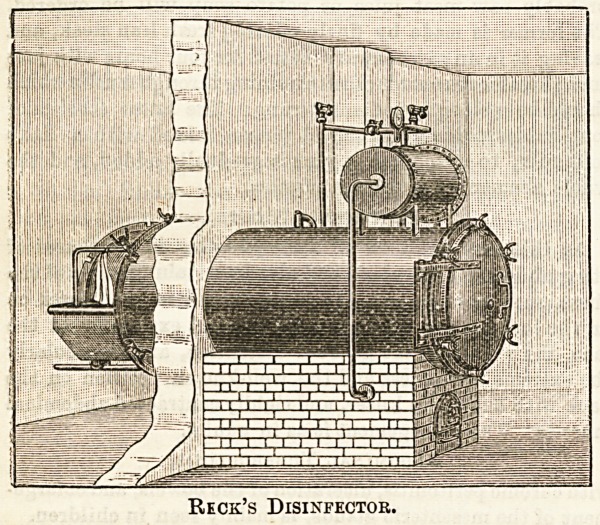


**Figure f2:**